# Blood Pressure Management Using the Hypotension Prediction Index During Paraganglioma Resection: A Case Report

**DOI:** 10.7759/cureus.83995

**Published:** 2025-05-12

**Authors:** Airi Akashi, Yohei Ono, Tetsuya Shimada, Tetsuya Takahashi, Takahiko Ikeda

**Affiliations:** 1 Anesthesiology, National Defense Medical College, Tokorozawa, JPN; 2 Biostatistics and Bioinformatics, St. Luke's International University Graduate School of Public Health, Tokyo, JPN; 3 Outcomes Research Consortium, University of Texas Health Science Center at Houston, Houston, USA; 4 Clinical Research Center, National Hospital Organization Murayama Medical Center, Musashimurayama, JPN

**Keywords:** blood pressure, cross-correlation analysis, hypotension prediction index, intraoperative hypotension, paraganglioma

## Abstract

Pheochromocytomas and paragangliomas are uncommon neuroendocrine tumors that present notable anesthetic challenges, especially in controlling blood pressure. The Hypotension Prediction Index (HPI) can be used for intraoperative hemodynamic monitoring and management. Here, we report the case of a 46-year-old woman diagnosed with paraganglioma who underwent laparoscopic retroperitoneal resection guided by a monitoring system with HPI. Overall, mean arterial pressure (MAP) and HPI displayed an inverted relationship during the anesthesia induction period and subsequent phases of the procedure. Her MAP, initially around 90 mmHg, reached 120-150 mmHg while intubated and during tumor manipulation, and thus intermittent doses of phentolamine were necessary to control hypertensive events. MAP then declined following ligation of the tumor’s feeding vessels, accompanied by a corresponding rise in HPI values. Intraoperative hypotension was managed by HPI-based protocols, resulting in a very low time-weighted average-MAP (TWA-MAP) below 65 mmHg of 0.007 mmHg. While the minimum cross-correlation value between HPI and MAP occurred at a time lag of zero, indicating no delay between HPI and MAP, we found HPI alerted 3 to 17 minutes before MAP reached below 65 mmHg. We could add a new insight into the interpretation of cross-correlation analysis, that no time delay between HPI and MAP might not necessarily mean the predictive ability of HPI was low. Whereas this case highlights the potential of the HPI in mitigating intraoperative hypotension, future research is necessary to evaluate its predictive accuracy during paragangliomas resection.

## Introduction

Pheochromocytomas (adrenal) and paragangliomas (extra-adrenal), which are generally grouped together into “pheochromocytoma-paraganglioma tumors (PPGLs)”, are uncommon neuroendocrine tumors arising from chromaffin cells of the autonomic nervous system. A recent systematic review estimates their incidence to range between 0.04 and 0.95 cases per 100,000 person-years [1]. Despite improvements in imaging techniques and catecholamine metabolite testing that have enhanced incidental detection [1], PPGLs remain rare, and clinical experiences with paraganglioma patients, particularly, are infrequent. Epidemiological data indicate that PPGLs occur more frequently in women and are more common in older adults [2]. Around 85%-90% of PPGLs are located in the adrenal glands and are referred to as pheochromocytomas, whereas the remaining 10%-15% arise outside the adrenal glands and are classified as paragangliomas [3].

Although treatment strategies for PPGLs vary depending on a patient’s symptoms, surgical resection remains the primary and most definitive approach [4]. One of the major anesthetic challenges in surgery is managing blood pressure. Patients with PPGLs typically exhibit severe hypertension before paraganglioma resection, arising from catecholamine release, followed by a dramatic decrease in blood pressure following the resection [4,5]. Because of the limited experience in anesthesia management for the rare operations of PPGLs, refractory hypotension after tumor resection may be severe and prolonged. As a result, hypoperfusion-related perioperative complications, potentially including intraoperative arrest [6] and postoperative myocardial injury [7], have been reported in patients with PPGLs. Furthermore, given that recent research has linked intraoperative hypotension to an increased risk of postoperative complications, including mortality [8], anesthesiologists must minimize its occurrence. Therefore, avoiding intraoperative hypotension is strongly recommended [9].

The Acumen Hypotension Prediction Index (HPI) software (Edwards Lifesciences, Irvine, CA), approved by the Food and Drug Administration, is designed to aid in preventing perioperative hypotension by providing early warnings to alert anesthesiologists about impending drops in blood pressure. HPI greater than 85 suggests that hypotension, defined as a mean arterial pressure (MAP) below 65 mmHg sustained for at least one minute, would occur within a median of four minutes after its warning [10]. A multinational study involving over 700 patients demonstrated that the use of the HPI significantly reduced both the severity and duration of intraoperative hypotension in patients undergoing non-cardiac surgery [11]. Conversely, there is currently a lack of evidence assessing whether HPI can effectively predict and mitigate intraoperative hypotension specifically following paraganglioma resection. In this case report, we present a patient with paraganglioma whose intraoperative hypotension was successfully managed with the aid of HPI.

## Case presentation

A 46-year-old asymptomatic woman presented with an abdominal mass, which was accidentally found during regular medical checkups. Abdominal ultrasound identified a 6 cm mass located near the right kidney, and subsequently, iodine-123 metaiodobenzylguanidine (^123^I-MIBG) scintigraphy demonstrated increased radiotracer uptake in the same region. Her medical history included a diagnosis of panic disorder for which she had previously received treatment. Preoperative laboratory findings, including blood and urine tests, electrocardiogram, and chest radiographs, were largely unremarkable, except for elevated serum metanephrine, serum normetanephrine, and urinary metanephrine levels. Her plasma free metanephrine and normetanephrine concentrations were 47 pg/mL and 1,580 pg/mL, respectively, yielding a metanephrine fraction of approximately 2.9% and classifying the tumor as noradrenergic. Based on these blood and urinalysis findings, as well as the uptake of ^123^I-MIBG in the tumor, a diagnosis of paraganglioma was made. Her history of panic disorder thus raised the possibility that symptoms might have been related to previously undiagnosed catecholamine excess. She was scheduled to undergo surgical resection of the paraganglioma with a laparoscopic retroperitoneal approach two months after diagnosis. One month before surgery, she was started on doxazosin at a daily dose of 4 mg to prevent perioperative blood pressure perturbations.

A combined general and epidural anesthesia approach at the T8-T9 level was planned for the operation. Although previous studies have reported that the combination of general and epidural anesthesia for PPGLs is associated with an increased incidence of perioperative hypotension [12], the decision to use epidural anesthesia was made based on the patient’s strong request. In addition to standard monitoring, such as a non-invasive blood pressure cuff, an intra-arterial line equipped with the Acumen^TM^ IQ sensor and HemoSphere monitoring platform (Edwards Lifesciences) was placed before induction. This setup enabled the use of the HPI and other hemodynamic parameters to help anticipate and prevent intraoperative blood pressure instability [13]. General anesthesia was induced with propofol and remifentanil and maintained using 1% sevoflurane in combination with remifentanil. A central venous catheter was then inserted as a precautionary measure to allow for the administration of high-dose vasoactive agents in the event of severe hypotension and hypertension. Before the surgical incision, 6 mL of 0.25% levobupivacaine and 100 μg of fentanyl were given through the epidural catheter.

The intraoperative trend of MAP and HPI throughout the surgery is illustrated inFigure 1 and Table 1. Notably, MAP and HPI displayed an inverted relationship during the induction period and subsequent phases of the procedure. Her MAP had increased immediately after endotracheal intubation and during tumor manipulation. To control these hypertensive surges, intermittent doses of phentolamine (0.5-1.0 mg per dose) and a continuous infusion of landiolol (3-5 μg/kg/min) were administered, along with a temporal increase in sevoflurane concentration to 1.5%-2.0%.

**Figure 1:**
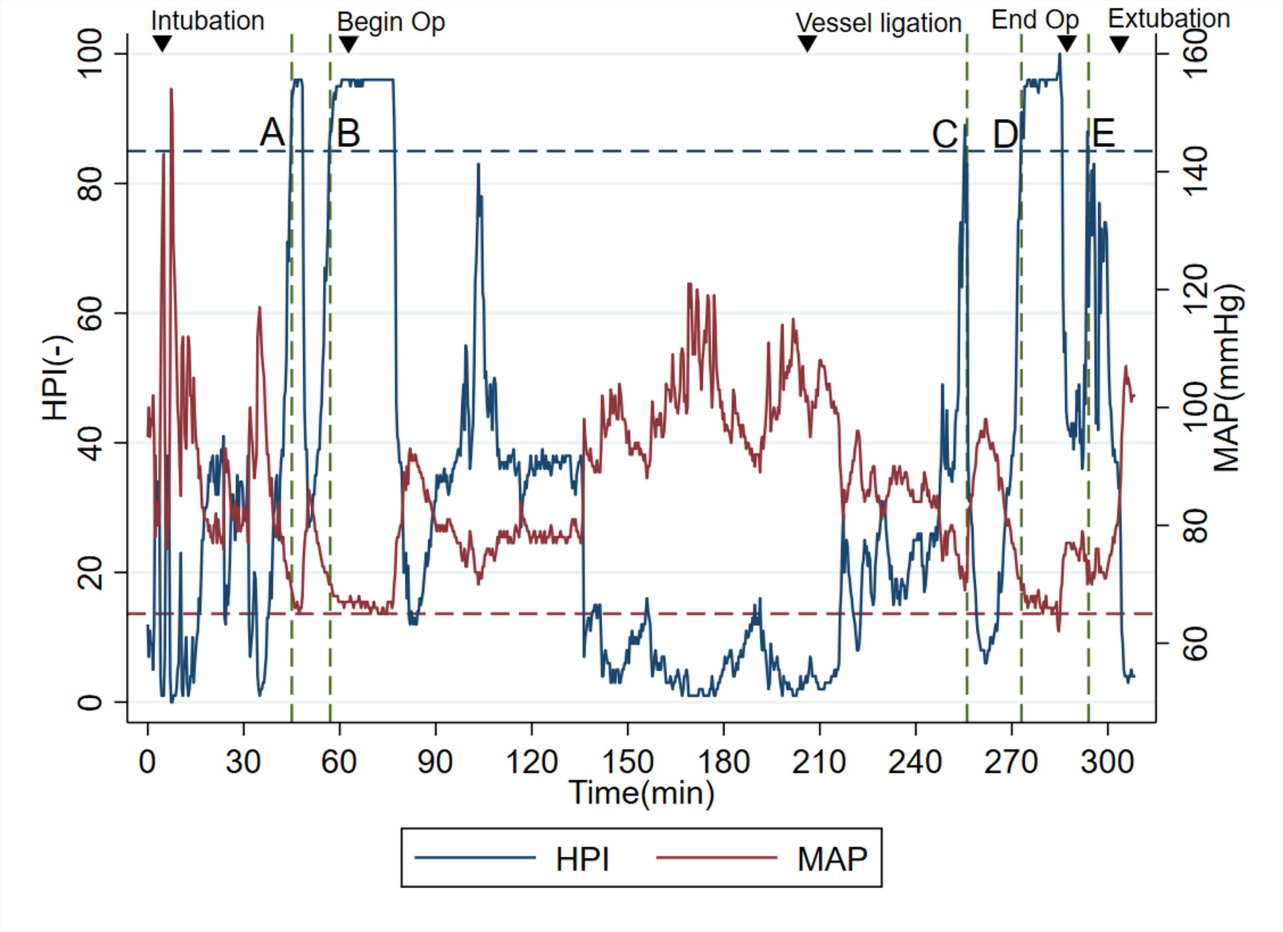
The overall trend of intraoperative mean arterial pressure (MAP) and the Hypotension Prediction Index (HPI) during the surgery. A horizontal dashed line with blue color represents the HPI of 85, the threshold value of hypotensive warning. A horizontal dashed line with red color represents the MAP of 65, regarded as the threshold value of intraoperative hypotension. Vertical green dashed lines with letters A to E represent the times when HPI reached greater than 85. Inverted triangles represent the time of events listed below: Intubation: endotracheal intubation; Begin Op: beginning of operation; Vessel ligation: ligation of the tumor’s feeding vessels; End Op: end of operation; Extubation: endotracheal extubation

**Table 1:** The overall trend of intraoperative hemodynamic parameters with the Hypotension Prediction Index (HPI) during surgery. HR: heart rate; SBP: systolic blood pressure; DBP: diastolic blood pressure; MAP: mean arterial pressure Intubation: endotracheal intubation; Vessel ligation: ligation of the tumor’s feeding vessels; Extubation: endotracheal extubation

Parameters	Pre-anesthesia induction	Intubation	Beginning of operation	Tumor manipulation with the highest MAP	Vessel ligation	Post-vessel ligation with the lowest MAP	End of operation	Extubation	Threshold value related to intraoperative hypotension
HR (beats/min)	77	117	55	66	69	60	63	88	N/A
SBP (mmHg)	133	201	89	164	136	87	107	112	<80-90
DBP (mmHg)	74	108	56	93	80	50	62	68	N/A
MAP (mmHg)	97	154	68	119	101	62	77	87	<65
HPI (-)	9	0	95	1	4	97	43	25	>85

As anticipated, blood pressure declines following ligation of the tumor’s feeding vessels, accompanied by a corresponding rise in HPI values. Hypotension was managed using HPI-based protocols from a previous study [13]: (1) vasopressors of norepinephrine and/or phenylephrine were administered when the cause of hypotension was regarded as vasoplegia, (2) fluid bolus was given when hypovolemia was suspected, and (3) ephedrine was delivered when impaired contractility was assumed. Table 2 represents the time points when HPI exceeded the hypotensive harm threshold of 85, corresponding to events A to E in Figure 1, and the related information including MAP values, types of interventions performed after HPI alerts, and how long they were until hypotension did happen after the warnings, in each time point.

**Table 2:** The time points when Hypotension Prediction Index (HPI) exceeded the threshold of 85 with corresponding mean arterial pressure (MAP), interventions, and subsequent MAP drops below 65 mmHg. The time points A to E represent times when HPI exceeded the hypotensive harm threshold of 85, with information including MAP values at the times, interventions performed, time points when hypotension occurred after the alerts, and duration of time in minutes between the HPI alert and successive hypotensive events. Letters A to E correspond to the same time points in Figure 1. Time 0 in minutes represents the time when anesthesia induction started. NAD: norepinephrine

Time points	Time when HPI>85	MAP when HPI>85	Intervention when HPI>85	Time when MAP<65	Time between HPI>85 and MAP<65 (mins)
A	43	69	None	46	3
B	55	70	None	72	17
C	254	69	NAD infusion started/phenylephrine bolus given	Not happened	NA
D	271	69	NAD rate increased	278	7
E	292	70	Volatile anesthesia stopped before extubation	Not happened	NA

**Figure 2:**
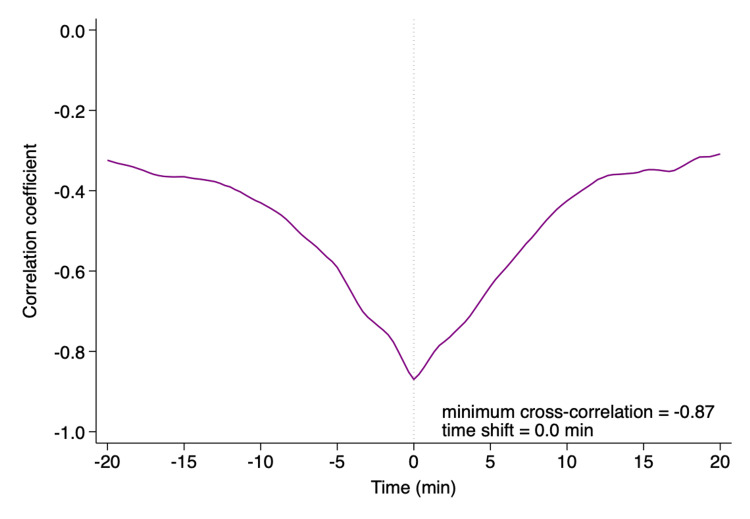
Cross-correlation analysis between the Hypotension Prediction Index and mean arterial pressure. A minimum cross-correlation of -0.87 at a 0.0 min time shift was observed.

The surgery was completed without major complications, aside from refractory hypotension, which required continuous norepinephrine infusion of up to 0.05 μg/kg/min to achieve MAP values of greater than 65 mmHg. The total duration of the surgery was 224 minutes, and the total infusion and urine output volumes were 2,050 mL and 770 mL, respectively. The time-weighted average (TWA)-MAP below 65 mmHg from induction to the end of anesthesia was 0.007 mmHg. In contrast, as shown in Figure 2, the maximum cross-correlation between MAP and HPI was observed with a 0.0 min time shift.

The norepinephrine infusion was generally reduced and discontinued on the first postoperative day. Even after the cessation of norepinephrine, the patient’s blood pressure remained stable without further hypotensive episodes. She was thus permitted to leave the intensive care unit on the same day. The post-surgical course was uneventful, without the new incidence of acute kidney injury, for instance, and she was discharged on the seventh postoperative day. At a follow-up outpatient visit one month after surgery, the patient was found to be making good progress in recovery without any major issues.

## Discussion

In this report of a patient undergoing the resection paraganglioma, we use HPI to avoid intraoperative hypotension. TWA-MAP below 65 mmHg during the surgery with HPI guidance was 0.007 mmHg. Compared to the previous study using HPI, where the median TWA-MAP below 65 mmHg was 0.03 (interquartile range: 0.00-0.20) mmHg, which was evaluated as very low [11], we can infer that HPI mitigated the negative effects of hypotension during paraganglioma resection. Dramatic blood pressure perturbations are common during the resection of PPGLs, and anesthesiologists need to intervene in a timely and appropriate manner. The latest PeriOperative Quality Initiative international consensus statement on perioperative arterial pressure management recommended avoiding hypotension when intraoperative hypertension is treated, as well as keeping intraoperative MAP greater than 60 mmHg in at-risk patients [9]. Our case with blood pressure disturbance was treated based on this consensus using HPI guidance. To the best of our knowledge, this is the first report of successful hypotension prevention using HPI during a specific type of surgery.

Preoperative assessment regarding features of targeted PPGLs is crucial for anesthesiologists to well prepare for the potential hemodynamic perturbations. For example, biochemically, whereas noradrenergic tumors, with metanephrine fraction less than 5%, may be complicated by hypotension “after” removal, patients with adrenergic features, with the fraction of greater than 5%, may suffer from hypotensive events even “before” the resection [14,15]. Furthermore, it was reported that larger tumor size of pheochromocytoma, another type of PPGLs, was associated with increased vasopressor requirement postoperatively, which was likely needed to relieve the hypotension after removal of the tumor [16]. It may be thus suggested that the resection of PPGLs with larger size could be a good target to use HPI to avoid severe perioperative hypotension, although the harm threshold of tumor size is unknown.

Whereas several trials suggested that hemodynamic management with HPI guidance reduced intraoperative hypotension [11,13], others did not [17]: A previous randomized trial with patients undergoing moderate- to high-risk noncardiac surgery showed that HPI failed to reduce intraoperative hypotension [17]. However, it was found that 55% of total alerts for future hypotension by HPI did not lead to actual intervention in the trial. Furthermore, the hypotension was 57% smaller when analysis was restricted to episodes in which clinicians intervened based on its guidance. A corollary is that the immediate intervention after the alert by HPI was critical to successfully prevent hypotension. The low value of TWA-MAP below 65 mmHg in this case suggests that our anti-hypotensive interventions with HPI were quick enough to prevent upcoming hypotension during the surgery.

Previous studies suggested that the concurrent MAP may predict future hypotension equally to the HPI [18,19]. In this context, an observational pilot study of 33 patients having high-risk noncardiac patients showed that there was no time delay between HPI and MAP, with the minimum cross-correlation of 0.0±0.0 minutes time shift for all patients, based on results of cross-correlation analysis [20]. The cross-correlation analysis is a statistical procedure that examines how trends in each variable synchronize over time. In our case, the minimum cross-correlation value between HPI and MAP also occurred at a time lag of zero, indicating no delay between HPI and MAP. The increased value of HPI in advance of the reduction of MAP allows anesthesiologists to intervene to prevent intraoperative hypotension. If the previous study is true [20], a high correlation between HPI and MAP in our case may indicate limited predictive value of HPI during the resection of paraganglioma.

In contrast, the cross-correlation analysis cannot discriminate the extent to which MAP would decrease and HPI would increase in each synchronized episode. For instance, time points from A to E in Figure 1 and Table 2 represent times when HPIs exceeded the hypotensive harm threshold of 85. First, at time points A and B, no anti-hypotensive interventions were performed because the operation was supposed to start soon, and potential hypertension derived from catecholamine secretion was of great concern. In this setting, HPI predicted hypotension 3 and 17 minutes before the events, respectively. As no interventions were performed at each time point, we might observe the pure ability of HPI to predict future hypotension. Second, at time points C and E, immediate interventions with vasopressor or cessation of volatile anesthesia were performed, resulting in no successive hypotensive events. They might demonstrate examples of successful anti-hypotensive treatments guided by HPI. And finally, at time point D, her MAP reached 65 mmHg, 7 min after HPI became 85, possibly due to insufficient treatment. Based on these clinical findings, except for time point D, we might suggest that the prediction by HPI could be helpful to reduce the severity and duration of intraoperative hypotension.

This case report has several limitations. First, anesthesiologists managing blood pressure during this case were not blinded. It might contribute to the fact that TWA-MAP below 65 mmHg of 0.007 mmHg in our case was very low, compared to that of the previous studies [11]. Future randomized controlled trials are needed to confirm whether HPI use accurately predicts and prevents perioperative hypotension and helps mitigate its severity during PPGL resection. Second, it is unclear how much this particular patient benefited from the hypotension avoidance management. Due to the lack of randomized trials and prospective analyses, whether an intraoperative hypotension avoidance strategy with HPI guidance would contribute to the reduction of the incidence of postoperative complications remains unknown, with contradictory results [21,22]. Furthermore, previous research indicated that the negative effect of intraoperative hypotension can vary depending on a patient’s background [23]. Therefore, we cannot determine the extent of the clinical benefit that our relatively healthy young patient gained from our HPI-based hypotension prediction strategy. Third, this is a preliminary report examining the effectiveness of HPI in an Asian young female diagnosed with a rare paraganglioma, and it remains unclear whether these findings apply to other populations with the same condition.

## Conclusions

To the best of our knowledge, we presented the first report of a patient undergoing paraganglioma resection whose potential blood pressure perturbation was managed using HPI software. HPI in this scenario appeared to support real-time blood pressure management, as evidenced by a very low TWA-MAP below 65 mmHg. Furthermore, we added a new insight regarding the interpretation of cross-correlation analysis: no time delay between HPI and MAP, in the context of “trend”, may not necessarily mean that the predictive ability of HPI is low. While our case illustrates the potential of HPI for intraoperative hypotension prevention, additional research is required to assess its role in optimizing perioperative hemodynamics in PPGL surgeries.
